# Type VI Choledochal Cysts—Case Report and Review of Literature

**DOI:** 10.1055/s-0039-1693652

**Published:** 2019-08-23

**Authors:** J. M. V. Amarjothi, Villalan Ramasamy, Jeyasudhahar Jesudasan, O. L. NaganathBabu

**Affiliations:** 1 Department of Surgical Gastroenterology, Madras Medical College, Rajiv Gandhi Government General Hospital, Chennai, Tamilnadu, India

**Keywords:** choledochal cyst type VI, cystic duct cyst, cholecystectomy

## Abstract

Choledochal cysts (CDC), are rare congenital dilations involving the extra hepatic biliary apparatus with or without dilation of the intrahepatic bile ducts. They are conventionally classified into five types. A new type, type VI, causing dilation of the cystic duct between the neck of the gall bladder and the common hepatic duct (CHD) has been described in medical literature which is the rarest of all these subtypes. They are commonly observed in middle aged females and are mostly symptomatic. Most of these cysts need magnetic resonance cholangiopancreatography (MRCP) for accurate diagnosis. Treatment options for these lesions are not well defined but range from simple cholecystectomy to complete excision of the entire bile duct and biliary reconstruction, as there is a concern of malignant transformation in these cysts. Hence, these rare cysts, though rare, must be borne in mind when dealing with suspicious cystic lesions in the biliary tract. Here, we present an interesting case of such a rare cyst and its management in a middle aged woman.

## Case Report

**Fig. 1:**
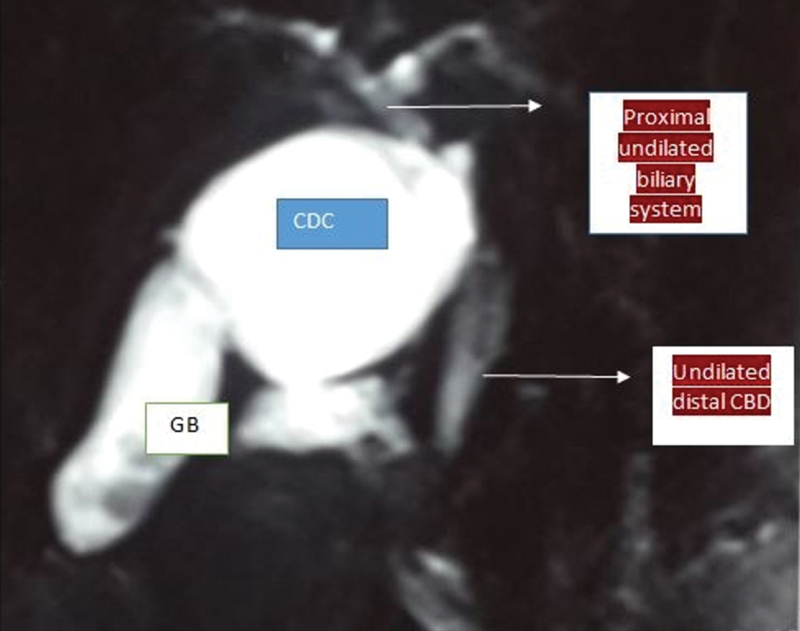
MRCP showing the contracted distal gall bladder (GB) with proximal cystic duct cyst or type VI choledochal cyst extending upto the undilated common bile. CDC, choledochal cyst; MRCP, magnetic resonance cholangiopancreatography.

**Fig. 2:**
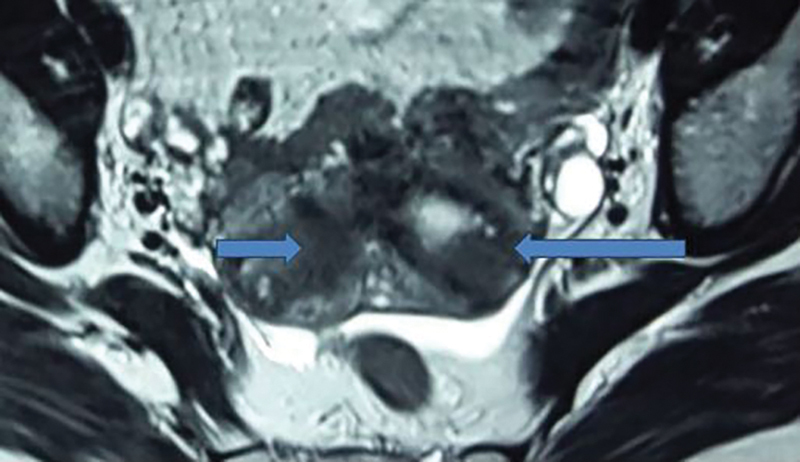
MRCP showing bicornuate uterus in same patient. (arrows) MRCP, Magnetic resonance cholangiopancreaticography.

A 40-year-old female presented with complaints of pain in the right side of upper abdomen which was aggravated by fatty meals for 6 months of duration. Clinical examination of abdomen and laboratory tests were normal. Ultrasound of the abdomen revealed a hypoechoic lesion of approximately 3 cm in size in close proximity to a thickened gall bladder (GB) with few stones in fundus .There was no intrahepatic biliary radicle dilatation (IHBRD). Magnetic resonance cholangiopancreatography (MRCP) revealed a 3 cm × 4 cm cyst interposed between the GB and the common hepatic duct (CHD), with no IHBRD, CHD, or common bile duct (CBD) dilation ( Fig. 1 ). A provisional diagnosis of type II/type VI choledochal cyst was made. Patient also had an incidental congenital anomaly involving the uterus (bicornuate uterus) seen on magnetic resonance imaging (MRI; Fig. 2 ).

Due to concerns of adhesions and difficult dissection between the cystic duct cyst and CHD, which may result in incomplete cyst excision, open mini-cholecystectomy with cyst excision was planned instead of laparoscopic cyst excision and cholecystectomy.

Intraoperatively, the GB and cystic duct cyst were dissected free from the surrounding structures and the plane between the cystic duct cyst and CHD was clearly defined. As the communication between the cyst and CHD was small, cholecystectomy and complete cyst excision was done.

**Fig. 3:**
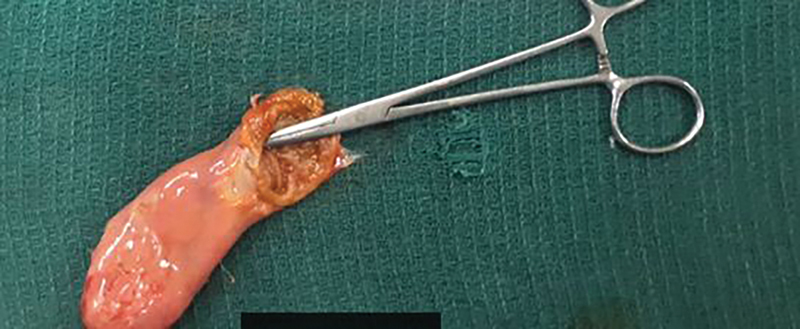
Post cholecystectomy specimen with opened proximal cystic duct cyst (forceps).

**Fig. 4:**
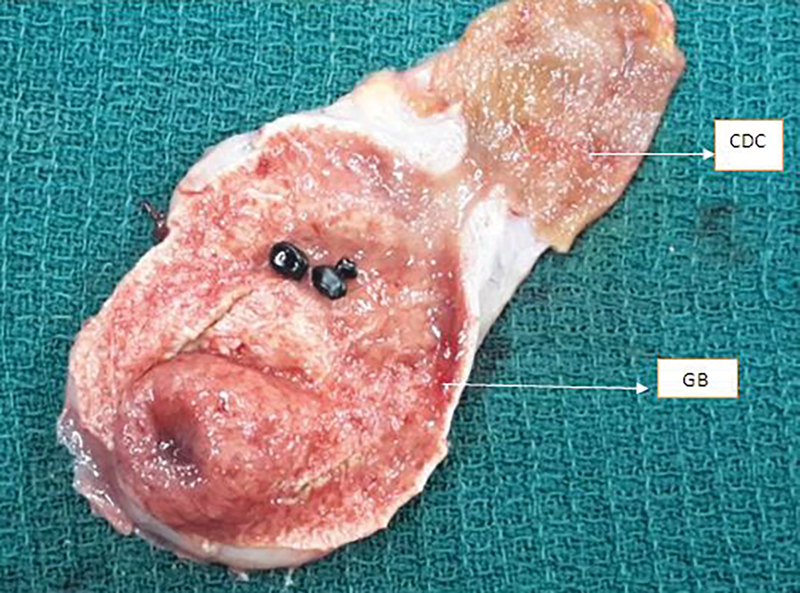
Post cholecystectomy specimen-longitudinal section showing proximal dilated cystic duct cyst and distal thickened gall bladder (GB) with small stones. CDC, choledochal cyst.

The postoperative course was uneventful and patient was discharged on postoperative day (POD) 3 after surgery. The specimen revealed a distal contracted GB with minute stones in fundus with cyst proximally ( Figs. 3 and 4 ). Microscopy of the GB showed features suggestive of chronic cholecystitis.

## Discussion

Choledochal cysts (CDC) commonly cause dilations in the extrahepatic bile ducts. They are classified by Todani et al 1 into five subtypes where type I CDC is the most common, (60%) causing fusiform dilation of the common biliary duct apparatus. Type II involves saccular diverticulum of the CBD, type III involves perivaterian part of CBD, type IV involves multiple focal dilations of the bile ducts which are further subdivided into extrahepatic with intrahepatic involvement (4a) and extra hepatic involvement only (4b). Type V involves the intrahepatic bile ducts only (Caroli's disease).

Serena Serradel et al 2 modified the widely accepted and used classification of Alonso–Lej which was previously modified by Todani et al 1 to include cystic duct cysts as a separate entity. Though the first such case was described by Bode and Aust in 1983, 3 these lesions are very rare and only a few cases have been described in literature ( Table 1 ).

**Table 1:** List of cases of type 6 choledochal cysts reported in literature

Author	No of cases	Year	Finding	Associated biliary anomalies	Diagnosis: intraoperative/preoperative (I/P)	Management
Bode and Aust 3	1	1983	Dilated cystic duct cyst with narrow neck	Cholangitis	I	Cholecystectomy, cyst excision, choledochoduodenostomy
Champetier et al 4	2	1987	Not known	Case 1: CBD cyst, case 2: cholelithiasis	P	Case 1: excision of cyst with bile duct cyst and cholecystectomy; case 2: excision of cyst with cholecystectomy
Serena Serradel et al 2	1	1991	Cystic dilatation of cystic duct	Cystolithiasis	I	Cholecystectomy, cystic duct excision
Loke et al 5	1	1999	Dilated cystic duct with wide opening into the CBD	Cystolithiasis	I	Cholecystectomy, cyst excision with RYHJ
Bresciani et al 7	1	1998	Cyst of cystic duct	Anomalous duct joining the cyst to right hepatic duct	I	Video laparoscopic en bloc resection of cyst and GB with ligature with a clip of the cystic duct and anomalous duct
Baj et al 8	1	2002	Fusiform dilatation, wide opening	NA	P	Patient refused surgery
Weiler et al 9	1	2003	Not known	APBDJ	P	Excision of cyst, CBD with cholecystectomy and RYHJ
Manickam et al 10	1	2004	Not known	APBDJ	NA	Excision of cyst with cholecystectomy
Yoon 11	3	2011	Case 1: fusiform dilatation of cystic duct; case 2: fusiform dilatation joining by a wide opening; case 3: fusiform dilatation with wide opening in the CBD	Case 1: advanced carcinoma GB with lymphadenopathy; case 2: fusiform dilatation of CBD; case 3: CBD dilatation, GB polyps	P	Case 1: not known; case 2: refused surgery; case 3: cyst excision, RYHJ
Chan et al 12	1	2009	Fusiform dilatation with narrow opening in CBD	Cholelithiasis, chronic Intraoperative	I	Laparoscopic excision of the cyst with cholecystectomy
Conway et al 13	1	2009	Fusiform dilatation with narrow opening in CBD	Intraoperative	I	Excision of cystic duct cyst with cholecystectomy
Ghatak 14	1	2010	Saccular dilatation	Fusiform dilatation of CBD Not known	NA	Excision of cyst, CBD, RYHJ ^l^
Khanna et al 15	1	2010	Cystic dilatation with wide opening into the common bile duct	–Dilation of CHD, CBD –Carcinoma gall bladder	P	Excision of cyst, gall bladder, and common hepatic duct with hepaticojeunostomy
De et al 16	1	2011	Cystic duct cyst with wide opening into CBD and normal distal CBD	Cholecystitis Intraoperative	I	Excision of cyst, gall bladder, and distal CBD, hepaticoenterostomy
Maheshwari 17	10	2012	Fusiform dilatation in six, saccular dilatation in four	1 case-fusiform CBD dilation 1 case-cystic duct calculi and malignancy	P	Surgical management of cyst: five cases, details of surgery not known Surgery for other indications, no of intervention for cystic duct cyst: 1 case; expectant management: 3 cases; refused follow-up: 1 case
Shah et al 18	1	2013	Cystic dilatation with wide opening	Cholecystitis	P	Excision of cystic duct and part of CBD with RYHJ
Mishra et al 19	2	2013	Case 1: fusiform dilatation with wide opening; case 2: fusiform dilatation of CBD with a wide opening	Case 1: CBD, diverticulum, Preoperative choledochocele, cholelithiasis; case 2: dilated CBD, right and left hepatic ducts, cholelithiasis	P	Case 1: excision of CDC with RYHJ ^l^ , deroofing of the choledochocele; case 2: CDC excision with RYHJ
Kesici et al 20	1	2013	Fusiform dilatation of cystic duct	Cholelithiasis	P	Elective excision of GB and cystic duct cyst
Sethi et al 21	3	2015	Case 1: cystic dilatation of cystic duct with wide opening; case 2: fusiform dilatation of cystic duct with wide opening; case 3: cystic dilatation of cystic duct with narrow opening	Case 1: carcinoma gall bladder; case 2: fusiform dilatation of hepatic duct; case 3: fusiform dilatation of both hepatic dust and common hepatic duct	P	Case 1: cholecystectomy with cystic duct cyst excision, removal of CBD with RYHJ; case 2: cholecystectomy with cystic duct excision, and CBD excision with RYHJ; case 3: open cholecystectomy with complete excision of extra hepatic biliary ducts with RYHJ with right and left hepatic ducts separately
Çamlıdağ et al 22	1	2015	Fusiform dilatation of the cystic duct with the CBD; cholangiocarcinoma in distal part of both cystic duct and CBD		P	Whipple's operation
Nambiar et al 23	1	2016	Fusiform dilatation of the cystic duct with GB with distal CBD including intrapancreatic portion		P	Lap converted to open cyst excision with cholecystectomy with hepaticojejunostomy
Ray et al 24	1	2017	Fusiform dilation of cystic duct with no IHBR		P	Laparoscopic cholecystectomy
UpadhyayaVD 28	3	2018	dilated cystic duct (3)	dilated CBD(3)	I(3)	Cyst excision with RYHJ(3)
This **case**		2019	Fusiform dilation of cystic duct with no IHBR		P	Open cholecystectomy

Abbreviations: APBDJ, abnormal pancreaticobiliary duct junction; CBD, common bile duct; CDC, choledochal cysts; GB, gall bladder; IHBR, intrahepatic biliary radicle; N/A, not available; RYHJ, Roux-en-Y Hepaticojejunostomy.

Most of these cystic duct lesions are symptomatic with most common symptom being epigastric and/or right upper quadrant pain aggravated by a fatty meal (as in this case). Although the exact etiology of these cysts is unknown, type VI CDC is thought to occur due to ectasia at the cystic duct caused by an abnormal pancreaticobiliary duct junction (APBDJ). 3 7 13 16 An abnormal APBDJ is, however, not seen in all cases and a focal aganglionosis of the cystic duct, such as seen in Hirschsprung's disease, is thought to play a role. 25

Abdominal ultrasonography is commonly the initial investigation and an MRCP is ideal to delineate the entire biliary system including the course of the cystic duct, presence or absence of ABBDJ, GB thickening, presence of gall stones, IHBRD, and CBD involvement. Endoscopic retrograde cholangiopancreatography (ERCP) is invasive, though providing the same information and detail regarding the biliary system as MRCP. ERCP and Tc-99m Hydroxy Imino Diacetic Acid (HIDA) scan can be used for diagnosis but are not commonly used.

Typical radiologic abnormalities that are specific to type VI CDC includes dilatation and squaring of the cystic duct, acute angulation of the CHD, and cystic duct junction with a distinct plane present between the dilated cystic duct and CHD, a normal or wide (Mirrizi's syndrome) opening of the cystic duct to the CBD, a normal CBD, and associated APBDJ. 26 Most common differential diagnosis is a type II or type I CDC due to similarities in appearance of cyst in close proximity to CBD. Type VI choledochal cysts can further be described based on morphology as fusiform (more common) and saccular.

As the epithelium of these cysts are prone to develop biliary intraepithelial neoplasia (BIN), onus must be placed on complete surgical excision and multiple cut sections of the histopathology specimen must be analyzed. 6 This is the rationale for complete surgical excision of the cyst along with cholecystectomy. 27

Hence, the treatment for symptomatic cystic duct cysts is cholecystectomy with complete excision of the cystic duct cyst. 1 5 For cysts with narrow opening of the cystic duct cyst into CHD, cholecystectomy with complete cystic duct excision alone would suffice and it can be done through laparoscopy by clipping the cyst opening into the CHD. 7 16 However, if the communication between the cystic duct cyst and CHD is wide, with adhesions precluding safe clipping, an open cyst excision along with Roux-en-Y hepaticojejunostomy as reconstruction may be performed. 7 16

Bresciani et al, 7 Chan et al, 12 and Ray et al 24 have reported on the laparoscopic management of the cystic duct cysts where the most common surgery is a laparoscopic cholecystectomy with cystic duct cyst excision. Laparoscopic cholecystectomy with cyst excision can be done with low threshold for conversion to open cholecystectomy in case of anatomical difficulty and associated biliary anomalies which are seen in most reported cases in literature. ( Table 1 )

## Conclusion

The increasing use of MRCP to diagnose hepatobiliary problems will result in an increasing number of such cystic duct dilations in the near future. In today’s laparoscopic era, many surgeons may also be faced with such cysts intraoperatively when they are operating on cases of acute cholecystitis or symptomatic biliary cholelithiasis. Hence, knowledge of type VI CDC, its diagnosis by MRCP, and treatment options are the need of the hour for effective treatment and management of this rare entity.
